# Safety and efficacy of microwave ablation for abdominal wall endometriosis: A retrospective study

**DOI:** 10.3389/fsurg.2023.1100381

**Published:** 2023-04-18

**Authors:** Yujiang Liu, Wanwan Wen, Linxue Qian, Ruifang Xu

**Affiliations:** Department of Ultrasound, Beijing Friendship Hospital, Capital Medical University, Beijing, China

**Keywords:** abdominal wall endometriosis, microwave ablation, interventional ultrasonography, ultrasound, contrast-enhanced ultrasound (CEUS) imaging

## Abstract

**Objectives:**

This retrospective study aimed to evaluate the safety and feasibility of ultrasound-guided microwave ablation in the treatment of abdominal wall endometriosis (AWE).

**Background:**

AWE is a rare form of endometriosis that often results in cyclic abdominal pain. The current treatment algorithm for AWE is not well established. Microwave ablation technology is a promising new thermal ablation technique for treating AWE.

**Methods:**

This was a retrospective study of nine women with pathologically proven endometriosis of the abdominal wall. All patients were treated with ultrasound-guided microwave ablation. Grey-scale and color Doppler flow ultrasonography, contrast-enhanced ultrasonography, and MRI were used to observe the lesions before and after treatment. The complications, pain relief, AWE lesion volume, and volume reduction rate were recorded 12 months after treatment to evaluate the treatment efficacy. Complications were classified according to the Common Terminology Criteria for Adverse Events and the Society of Interventional Radiology classification system.

**Results:**

Contrast-enhanced ultrasound showed that all lesions underwent successful treatment with microwave ablation. The average initial nodule volume was 7.11 ± 5.75 cm^3^, which decreased significantly to 1.85 ± 1.02 cm^3^ at the 12-month follow-up with a mean volume reduction rate of 68.77 ± 12.50%. Periodic abdominal incision pain disappeared at 1 month after treatment in all nine patients. The adverse events and complications were Common Terminology Criteria for Adverse Events grade 1 or Society of Interventional Radiology classification grade A.

**Conclusions:**

Ultrasound-guided microwave ablation is a safe and effective technique for the treatment of AWE, and further study is warranted.

## Introduction

Abdominal wall endometriosis (AWE), defined as the presence of endometrial glands and stroma in the abdominal wall, is a rare extrapelvic endometriosis (1, 2). Most cases of AWE are associated with uterine surgical procedures such as cesarean section delivery, hysterectomy, and amniocentesis (3). With the increase in the cesarean delivery rate, the number of AWE cases has shown an obvious upward trend (4). Although AWE is a nonmalignant disease, clinical symptoms are present and include a mass in the abdominal wall or a nodule at the previous scar, along with cyclic abdominal pain or progressively intensifying menstrual cramps that impact the patient's quality of life (5). Therefore, accurate diagnosis and effective treatment for AWE are important.

For AWE diagnosis, the most useful tools are ultrasound and magnetic resonance imaging (MRI) of the pelvis and abdomen (6). Computed tomography (CT) is a poor imaging modality because of the lack of resolution and radiation exposure. In addition, because of the rarity of AWE and the need for the pathological confirmation of diagnosis, the ultrasound-guided core-needle biopsy is a useful, noninvasive procedure for positive diagnosis and differential diagnosis (7).

Traditionally, AWE has been treated with wide surgical resection. However, in some cases, complete surgical resection can cause further operative trauma and require complex abdominal wall repair, flap placement, or mesh implantation (8). In addition to cyclic pain because of the hormonal changes across the endometrial cycle, hormonal therapy has been used for the treatment of AWE. However, the success rate of the use of medications has been reported to be low because medications offer only temporary alleviation of clinical symptoms (9, 10). Therefore, the need for an efficient nonsurgical method of removing AWE lesions is urgent.

Small studies have presented the use of minimally invasive techniques, including high-intensity focused ultrasound (HIFU) ablation and percutaneous image-guided cryoablation, to treat AWE (11, 12). There are some disadvantages to HIFU ablation, including nonpathological results and residual lesions in the abdominal wall (13). Percutaneous image-guided cryoablation is performed under general anesthesia and may cause additional complications (14, 15). Ultrasound-guided microwave ablation is characterized by high ablation temperatures, large ablation volumes, fast ablation times, and the ability to use multiple applicators simultaneously and can be proposed in outpatients (16). Sufficient hydrodissection technology could ensure safe, effective expanded ablation and reduce injury complications (17). Several studies have shown that microwave ablation therapy is safe and effective for the treatment of thyroid nodules (18, 19), hepatocellular carcinoma (20), and renal cell carcinomas (17). However, microwave ablation has not been widely carried out in AWE, and the long-term outcomes are still unknown. Therefore, this study aimed to evaluate the safety and feasibility of ultrasound-guided microwave ablation in the treatment of AWE in a single institution retrospective cohort.

## Methods

### Study design and patients

This was a retrospective, observational, and descriptive study that included nine patients with AWE who underwent ultrasound-guided microwave ablation treatment at Beijing Friendship Hospital from August 2014 to January 2021. All patients underwent ultrasonographic examinations, contrast-enhanced ultrasound (CEUS) screening, MRI, and ultrasound-guided core-needle biopsy before microwave ablation. The diagnostic criteria for AWE were as follows: (1) women of reproductive age with a history of cesarean section; (2) masses or nodules around the cesarean section scar with cyclic pain that is exacerbated during menstruation; (3) ultrasound showing nodules near the cesarean section scar; and (4) ultrasound-guided biopsy and hematoxylin–eosin staining revealing that endometrial glands or stroma in the AWE (6, 11). Data on the participants’ baseline demographics, including age, the number of cesarean incisions, type of cesarean section, the time interval from the last cesarean delivery, subjective symptoms regarding the texture of the lesion, cyclic or continuous pain, pain on palpation, and protrusion of the skin, were collected. Weight (kilograms) and height (meters) were measured using standardized and reproducible study protocols. Body mass index (BMI) (kg/m^2^) was calculated by the formula of weight divided by height squared.

### Preablation preparation

The patients who were taking anticoagulant drugs (aspirin or clopidogrel bisulfate) long-term were required to stop these medications at least 3–5 days before treatment and for 3 days after treatment. None of the patients received pharmacological therapy or underwent surgical therapy before the microwave ablation treatment. Grey-scale ultrasound and color Doppler flow imaging (CDFI) were performed using 3.5- and 5.0-MHz convex-array and 7.5-MHz linear-array transducers to assess the location, the three orthogonal diameters, the shape, the depth, the echotexture, the appearance of the margins, and blood flow of the nodules to be ablated. The three orthogonal diameters of the nodule included the maximum diameter (d1) and two vertical diameters (d2 and d3).Then, the volume of the nodule was calculated by the following equation: volume = π/6 × d1 × d2 × d3. The physicians performing the examination gave a subjective impression of scarce blood vessels when only one to five vascular spots (of arterial or venous origin) were found within the nodule and abundant blood vessels if more than five vascular spots could be seen (21).

To evaluate the range and blood perfusion of the nodule, CEUS screening was performed after a bolus of 2.4 ml SonoVue (Bracco Company, Milan, Italy) that was injected through a central line and followed by 5 ml of normal saline as a flush. A CEUS scan was performed with a mechanical index of ≤0.04. The following temporal characteristics of enhancement were detected by a chronometer included in the device: time between injection and appearance of microbubbles; the time between injection and disappearance of microbubbles; and duration of contrast enhancement. A single physician with 7 years of experience in ultrasonography performed and digitally recorded all CEUS scans.

MRI, as a sensitive tool to detect thermal lesions, may provide more accurate data about the penetration, extension, and type of content of the nodule. MRI of the pelvis was performed at 1.5 T (Magnetom Symphony, Siemens) using a six-channel pelvic phased-array coil with the capability of parallel imaging. The MRI examinations included fat-suppressed spin echo T1-weighted imaging and high-resolution turbospin echo T2-weighted imaging (22, 23). MRI images were interpreted by two radiologists with at least 7 years of experience in MRI images, and any discrepancy was resolved by consensus.

In addition, all nodules were examined through a preoperative ultrasound-guided core-needle biopsy to obtain the histopathological diagnosis of AWE. Iodine-alcohol was used to sterilize the region where the procedure was performed and the ultrasound probe was used to guide the procedure. We performed at least three biopsies under sonographic guidance to obtain enough tissue for representative histopathological evaluation. Each patient was kept under observation for 10–30 min after the aspiration procedure while firm, local compression was applied to the biopsy site. Hematoxylin–eosin staining was performed to analyze the morphological features of the AWE specimens. The histopathologic criteria for the diagnosis of endometriosis in our study included the following endometrial characteristics: stroma, endometrial-like glands, and hemosiderin pigment (21, 24). All ultrasound examinations and ultrasound-guided core-needle biopsy procedures were performed by experienced staff with 10 years of clinical experience in performing US examinations.

### Ablation procedure

A microwave ablation therapy instrument (KY-2000, Kangyou Medical, Nanjing, China) was used in this study, with a microwave generator (producing 1–100 W of power at 2,450 MHz), a flexible low-loss coaxial cable and a cooled shaft antenna (1.6 mm in diameter, 10 cm in length, and stable temperature at 28–32°C). The entire process was guided by a Hitachi Ascendus color Doppler ultrasound system with an L75-type high frequency linear-array transducer. After the equipment was well prepared, the microwave ablation procedure was performed in the operation room. Patients were placed in a supine position with a fully exposed abdomen, and then the operator prepared the surgical area and administered local infiltration anesthesia with 2% lidocaine through a 22G needle after sterilization. A safe distance between the nodule and the critical structure was maintained throughout the operation to protect the critical structures from thermal injury. For protection of the skin, a mixture of 2% lidocaine (10 ml) and 0.9% physiological saline (20 ml) was infused on the side close to the fat layer and skin to achieve a “liquid isolating region” before ablation. The isolation fluid was also applied in the deep surface of the lesions if necessary. For the mainly solid nodules, we performed microwave ablation directly; for the mixed/mainly cystic nodules, we aspirated the liquid before ablation. Under ultrasound guidance and dynamic monitoring, the pin of the microwave antenna was precisely inserted into the targeted nodules, and the microwave ablation treatment was initiated. The power of the microwave instrument was 30–50 W and the duration of ablation was 20–120 s. With the release of microwave energy, the echo from the microwave needle was enhanced and continued to expand. By multipoint mobile ablation, the generated hyperechoic area completely encompassed the entire AWE nodule, and CDFI showed no blood flow signal in the nodules. Then, the ablation procedure was terminated. The vital signs of the patients were intensively monitored during the whole process. The time duration, power, and any complications of the microwave ablation were recorded. All ablation procedures were performed by two physicians with more than 20 years of experience in abdominal interventional ultrasound.

### Post-ablation evaluation and follow-up

Immediately after the microwave ablation treatment, CEUS was performed to evaluate the response of the lesion to microwave ablation treatment. The non-perfused volume (indicative of successful ablation) and the non-perfused volume rate (defined as the non-perfused volume divided by the nodule volume) were observed in all patients immediately after treatment. After the procedure, all patients were monitored in the hospital for a minimum of 2 h. Any adverse events after treatment, such as pain, fever, skin thermalgia, infection in the treatment area, and actual or potential injury, were recorded and classified according to the Common Terminology Criteria for Adverse Events 5.0 (CTCAE) of the National Cancer Institute (25) and were defined as a major or minor complication according to the standardized grading system of the Society of Interventional Radiology (SIR) (26). All patients were asked to return to the outpatient department for a gray scale ultrasound, CDFI, and MRI examination 1 year after ablation. The size, echogenicity, and vascularity of the nodules were examined, and the volume was calculated. The treatment efficacy was evaluated according to the volume reduction rate (VRR), which was calculated using the following equation: VRR (%) = [(pretreatment volume − follow-up volume)/pretreatment volume] × 100%.

### Statistical analysis

Continuous variables were tested for normality of distribution by the Shapiro–Wilk test. Normally distributed variables are presented as the mean ± SD and were compared with paired-samples t-test; non-normally distributed variables are reported as medians (25th–75th percentile) and were compared with Wilcoxon signed rank tests (for asymmetrically distributed data). Categorical variables were expressed as numerals (percentages) and were compared with the chi-square test. A *p*-value <0.05 was considered statistically significant. Analyses were performed using SPSS version 25 software (SPSS Inc., Chicago, IL, United States).

## Results

### Patient characteristics

From August 2014 to January 2021, nine women (age: 33.67 ± 5.68 years, BMI: 25.68 ± 3.37 kg/m^2^) diagnosed with AWE were enrolled in this study. All patients had a history of cesarean section prior to the onset of AWE. All of the patients had one cesarean delivery. The type of the cesarean incision was transverse in 100% of the patients. The average interval time between the cesarean section and the first symptoms of abdominal wall nodules was 29.78 ± 18.20 months. The texture of the lesion was hard in 88.9% of the patients. The pain was reported to be cyclic in seven patients, and the pain increased during menses and seldom occurred between cycles. Pain upon palpation was experienced in 66.7% of the patients, and the others experienced no pain. Skin protrusion was seen in 66.7% of the patients. The patients’ clinical characteristics are shown in Table 1.

**Table 1:** Demographic characteristics of patients with AWE.

Variables	*n* = 9
Age, years	33.67 ± 5.68
BMI, kg/m^2^	25.68 ± 3.37
<18.5 (underweight)	0 (0.0)
18.5–24.9 (normal weight)	5 (55.6)
25–29.9 (overweight)	3 (33.3)
>30 (obese)	1 (11.1)
Number of cesarean incisions, *n* (%)	
One	9 (100.0)
Two	0 (0.0)
Type of cesarean incision, *n* (%)	
Vertical	0 (0.0)
Transverse	9 (100.0)
Time interval from the last cesarean delivery, months	29.78 ± 18.20
Subjective symptoms texture of the lesion, *n* (%)	
Hard	8 (88.9)
Soft	1 (11.1)
Cyclic or continuous pain, *n* (%)	
Cyclic	7 (77.8)
Continuous	2 (22.2)
Pain on palpation, *n* (%)	
Yes	6 (66.7)
No	3 (33.3)
Protrusion of the skin, *n* (%)	
Yes	6 (66.7)
No	3 (33.3)

AWE, abdominal wall endometriosis; BMI, body mass index.

Data are presented as mean ± SD or *n* (%), unless otherwise stated.

### Baseline imaging features of AWE

Ultrasound examination of the AWE showed the presence of a fixed solid hypoechoic mass (less echogenic than the surrounding hyperechoic fat) within the abdominal wall. All patients had a single nodule. In fact, the nodules appeared solid (only one patient had some cystic areas within the AWE). Before treatment, the mean nodule volume was 7.11 ± 5.75 cm^3^. On sonography, approximately 66.7% of the nodules were located along the scar of a cesarean section. Five lesions were confined to the subcutaneous tissue, two lesions involved the muscle layer, and two infiltrated both the superficial and deep layers. The nodules had a round/oval shape in 66.7% of the patients and a stellate morphology in 33.3% of the patients. The echotexture was heterogeneous in 77.8% of the nodules owing to the presence of small cystic lacunae or inner hyperechoic; in the remaining nodules, it was homogeneously hypoechoic. All nodules had ill-defined, blurred outer borders. CDFI examination revealed scarce blood vessels within the AWE in all patients. The sonographic characteristics of the nodules are shown in Tables 2, 3.

**Table 2:** Sonographic features of AWE lesions.

Variables	*n* = 9
Lesion volume, cm^3^	7.11 ± 5.75
Location, *n* (%)	
To the left of the scar	1 (11.1)
At the middle of the scar	6 (66.7)
To the right of the scar	2 (22.2)
Depth, *n* (%)	
Superficial (subcutaneous tissue only)	5 (55.6)
Deep (involving the muscle layer)	2 (22.2)
Involving superficial and deep layers	2 (22.2)
Shape, *n* (%)	
Round/oval	6 (66.7)
Stellate/irregular	3 (33.3)
Echotexture, *n* (%)	
Heterogeneous	7 (77.8)
Homogeneously hypoechoic	2 (22.2)
Margins, *n* (%)	
Ill-defined, blurred	9 (100.0)
Smooth	0 (0.0)
Vascularization at color Doppler, *n* (%)	
Scarce blood vessels	9 (100.0)
Abundant blood vessels	0 (0.0)

AWE, abdominal wall endometriosis.

Data are presented as mean ± SD or *n* (%), unless otherwise stated.

**Table 3:** Baseline characteristics of the AWE lesions.

Lesion	Location (scar)	Depth	Shape	Echotexture	Margins	Vascularization at CDFI	Size (cm)
1	Middle	Deep	Round/oval	Homogeneously hypoechoic	Blurred	Scarce	1.63 × 1.51 × 1.19
2	Middle	Superficial + deep	Stellate/irregular	Heterogeneous	Blurred	Scarce	1.50 × 1.40 × 1.30
3	Middle	Superficial	Round/oval	Homogeneously hypoechoic	Blurred	Scarce	2.94 × 1.30 × 1.05
4	Middle	Superficial	Round/oval	Heterogeneous	Blurred	Scarce	2.60 × 1.10 × 2.60
5	Right	Superficial + deep	Stellate/irregular	Heterogeneous	Blurred	Scarce	3.13 × 1.02 × 2.61
6	Middle	Superficial	Round/oval	Heterogeneous	Blurred	Scarce	2.53 × 2.97 × 1.12
7	Right	Superficial	Round/oval	Heterogeneous	Blurred	Scarce	3.00 × 3.57 × 3.03
8	Middle	Deep	Stellate/irregular	Heterogeneous	Blurred	Scarce	2.13 × 3.47 × 3.02
9	Left	Superficial	Round/oval	Heterogeneous	Blurred	Scarce	1.86 × 4.89 × 3.71

AWE, abdominal wall endometriosis; CDFI, color Doppler flow imaging.

**Figure 1:**
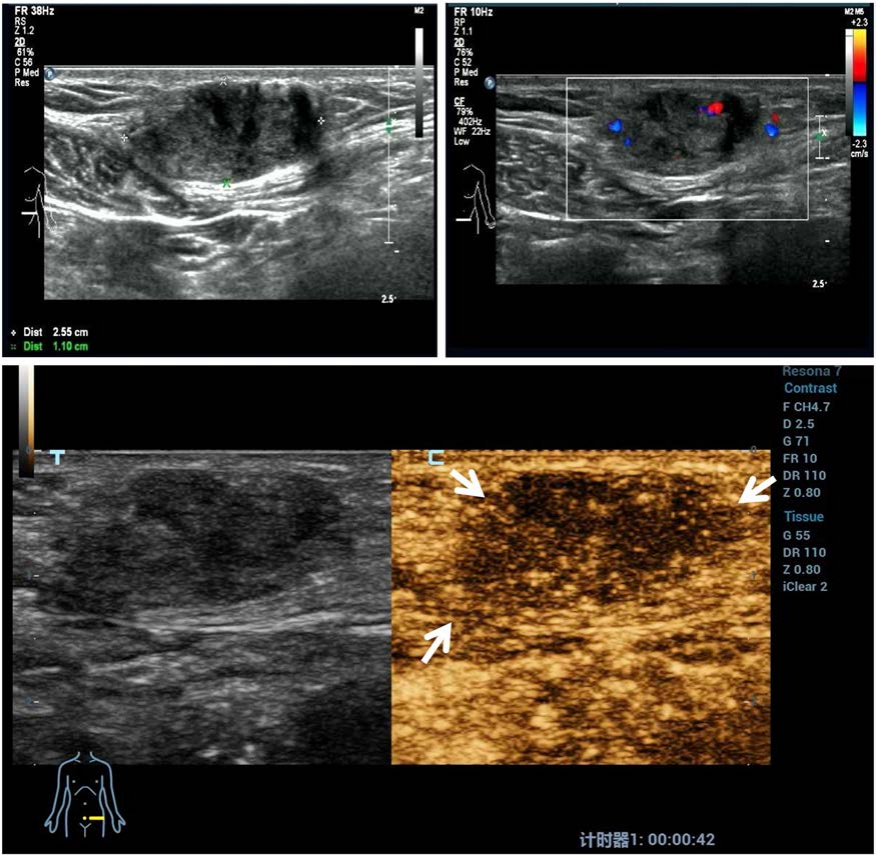
A representative case before ultrasound-guided microwave ablation. A 41-year-old patient had a hard mass on a transverse cesarean incision with cyclic abdominal wall pain. Ultrasound examination of the AWE mass revealed a heterogeneous mass with ill-defined, blurred outer margins. Color Doppler ultrasound images show scarce flow within the mass. The contrast-enhanced ultrasound imaging of the AWE nodules shows slight perfusion compared with the surrounding tissue. AWE, abdominal wall endometriosis.

Before the microwave ablation treatment, CEUS imaging of the nodules showed slight perfusion in all the nodules. A representative image is illustrated for CDFI and CEUS imaging before the microwave ablation procedure in Figure 1.

In addition, the AWE nodules showed remarkable enhancement on enhanced fat-suppressed T1-weighted images and inhomogeneous high-signal intensity on the T2-weighted images before the microwave ablation treatment.

**Figure 2:**
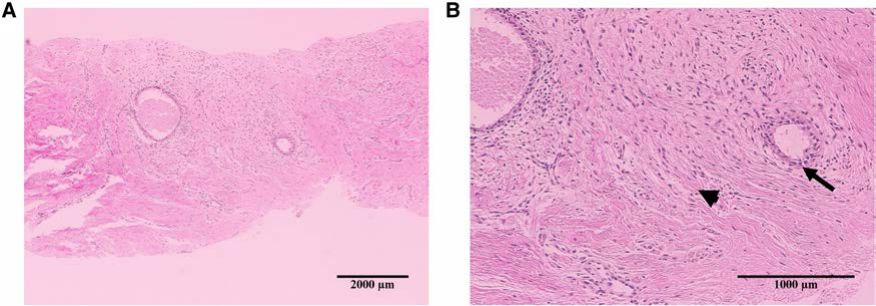
Representative images of hematoxylin–eosin staining in the histological specimen of abdominal wall endometriosis. The nodule shows scarce endometrial glands (arrowheads) and stroma (arrows) in the abdominal wall: 4× (**A**) and 10× (**B**).

AWE diagnoses were achieved by core-needle biopsy, and the diagnoses were characterized by the presence of scarce endometrial glands and spindle cell stroma embedded within fibroblasts, collagen fibers, and skeletal muscle cells. Hemosiderin deposition consistent with prior bleeding was present in the majority of the patients (Figure 2).

### Microwave ablation treatment evaluation

**Figure 3:**
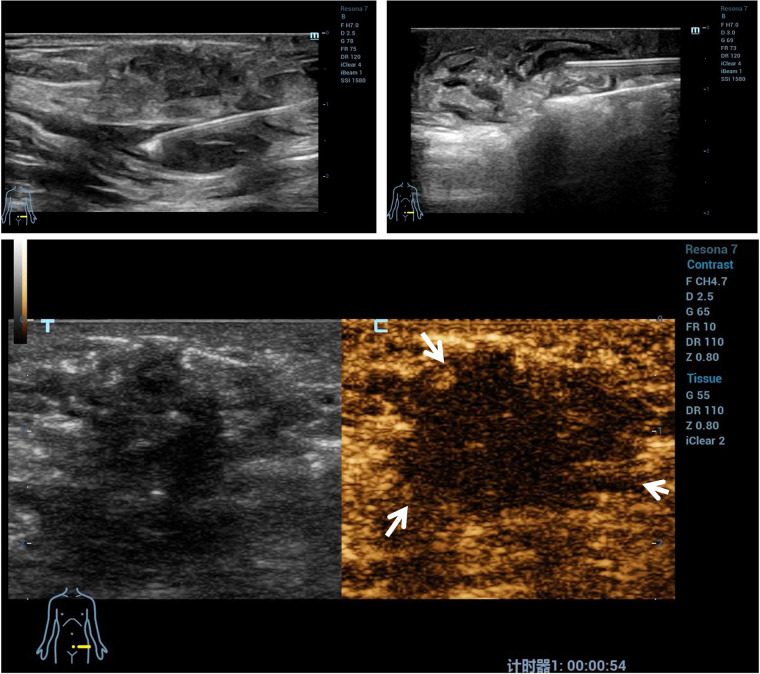
A representative case of microwave ablation treatment. Microwave ablation was performed, and a gasification reaction of the hyperechoic cloud was observed inside the lesion region. The contrast-enhanced ultrasound imaging of AWE nodules shows no contrast agent filling in the nodules. AWE, abdominal wall endometriosis.

As shown in Table 4, after treatment for 295.00 (169.00–509.50) s with a mean energy of 32.56 ± 5.13 W, the lesions within the blood flow signal disappeared as observed through the CDFI, and no contrast agent filled in the nodules, characterized by the ““black hole sign” as observed through CEUS, and this suggested that the blood perfusion in the nodules disappeared and that the ablation was completed. The non-perfused volume (indicative of successful ablation) was 3.40 (1.79–7.82) cm^3^ and the non-perfused volume rate was 68.26 ± 6.23% (Figure 3).

**Table 4:** Perioperative and postoperative evaluation of ablation for AWE.

Variables	*n* = 9
Non-perfused volume, cm^3^	3.40 (1.79–7.82)
Non-perfused volume rate, %	68.26 ± 6.23
Ablation power, W	32.56 ± 5.13
Ablation time, s	295.00 (169.00–509.50)
Adverse effects within 24 h post-procedure, *n* (%)	
Skin thermalgia	1 (11.1)
Infection in the treatment area	1 (11.1)
Lesion volume at 12 months post-procedure, cm^3^	1.85 ± 1.02
Volume reduction ratio at 12 months post-procedure, %	68.77 ± 12.50

AWE, abdominal wall endometriosis.

Data are presented as mean ± SD, median (interquartile range), or *n* (%), unless otherwise stated.

### Post-procedure evaluation

All patients were required to report any discomfort or adverse effects occurring within one day post-microwave ablation treatment and to return to our department at 12 months after ablation. After treatment, one patient reported mild skin thermalgia and one patient reported mild infection in the treatment region. The adverse events and complications were CTCAE grade 1 or SIR classification grade A. These patients recovered within 15 days post-treatment without any medication. No postoperative fever, skin burns, skin blistering, or other important structural complications were observed.

**Figure 4:**
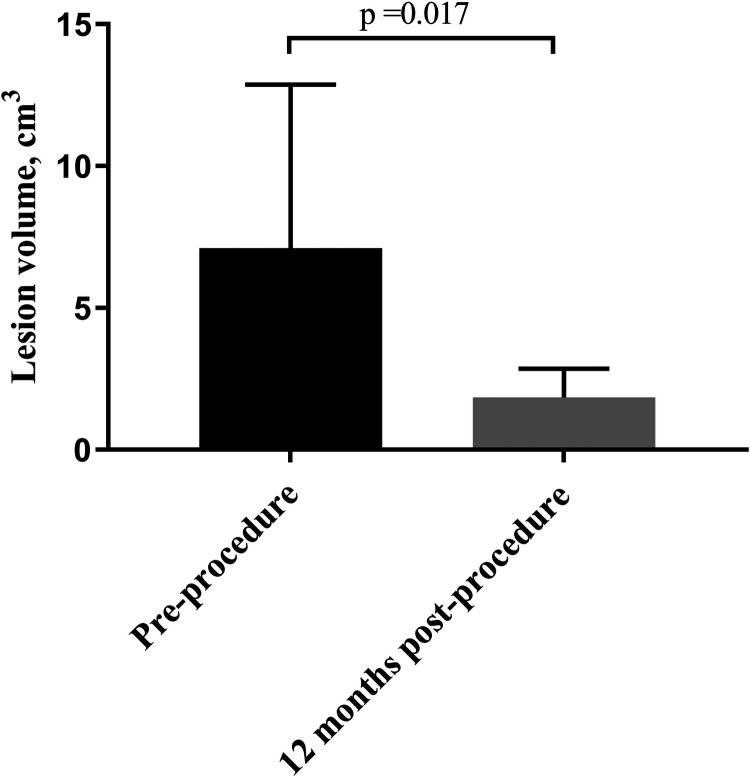
Comparison of pre- and post-procedure two-dimensional ultrasound evaluations of patients with AWE. Compared with the pre-procedure volume, the average volume of the AWE nodules was significantly decreased at the 12-month follow-up. AWE, Abdominal wall endometriosis.

**Figure 5:**
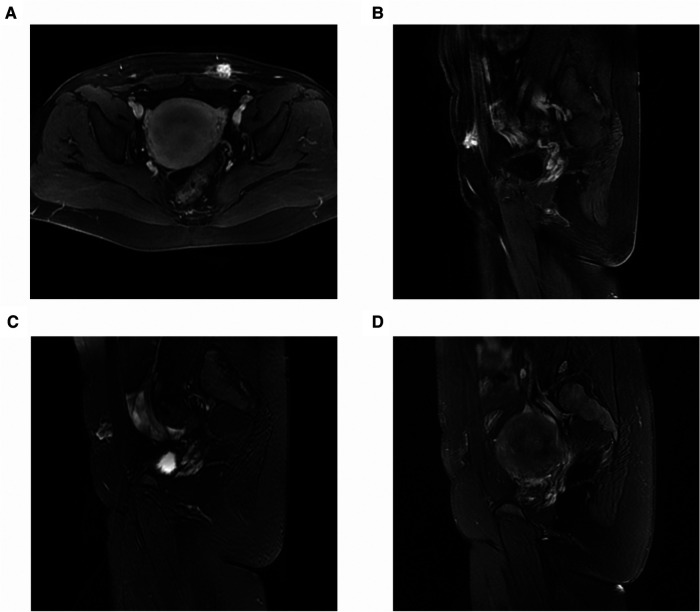
MRI of a 31-year-old patient with abdominal wall endometriosis treated with ultrasound-guided microwave ablation. Before microwave ablation, MRI showed remarkable enhancement on enhanced fat-suppressed T1-weighted axial images (**A**) and sagittal images (**B**), and inhomogeneous high-signal intensity on the T2-weighted sagittal images (**C**). After treatment, coagulation necrosis in the lesion area indicated that there was no residual enhancement at the treatment site and that the lesion had decreased in size (**D**). MRI, magnetic resonance imaging.

At 12 months of follow-up, no patient had periodic pain. The volumes of all the treated nodules had significantly decreased from 7.11 ± 5.75 cm^3^ pre-procedure to 1.85 ± 1.02 ml at 12 months (*P* = 0.017) (Figure 4). The VRR at 12 months post-procedure was 68.77 ± 12.50%. After treatment, coagulation necrosis in the lesion area showed no residual enhancement at the treatment site and had decreased in size (Figure 5).

## Discussion

In this study, we found that the volume of all AWE nodules significantly decreased after microwave ablation treatment. Furthermore, no major complications were observed after microwave ablation treatment. All patients had subjective improvement in their cyclical abdominal wall pain with menstruation. The current study demonstrates that ultrasound-guided microwave ablation is feasible and effective in the treatment of AWE.

Endometriosis is a common gynecological condition characterized by the presence of endometrial epithelial and stromal cells in non-uterine locations. These glands and stroma are typically pelvic but are also found in other locations, most commonly the bowel, diaphragm, umbilicus, and pleural cavity (3). The presence of endometriotic infiltration in the scar tissue of abdominal incisions, such as laparoscopic port sites, hernia repairs, and laparotomies, is collectively referred to as AWE (7). The pathogenesis of the AWE may be due to the direct implantation of endometrial epithelial and stromal cells into the soft tissues of the abdominal wall during abdominal-pelvic surgeries, particularly with the growing popularity of cesarean sections (6, 24, 27). The rates of AWE have been estimated to range from 0.03% up to 0.45% after cesarean section (7). Characteristic clinical features include a mass in the abdominal wall or a nodule at the previous scar and local pain at the cesarean scar/incision site of the abdominal wall during menstruation (28). Symptomatic AWE can result in long-term adverse effects on the quality of life and work productivity and can result in massive increases in healthcare costs. Therefore, it is extremely essential to diagnose and treat AWE in the early stage.

Although some evidence has demonstrated that the typical history, symptoms, and physical examination are sufficient to make a diagnosis of AWE (21, 28, 29), preoperative imaging may be necessary in patients who have an atypical clinical aspect, including patients who have no cyclic pain or modification of the nodule or with a malignant transformation of AWE. The most useful assessment tools to diagnose AWE are ultrasound, CT, and MRI of the abdomen. PET/CT is less useful because of the low metabolic rate of the cells. Gray scale US and CDFI usually represent the first step in the evaluation of soft tissue masses. It could be used to provide the location, the size, the shape, the depth, the echotexture, the margins, and the intralesional vascularization of AWE (30, 31, 32). The CEUS scan was also used to assess the extent and blood supply of the AWE (33). However, ultrasonography cannot reliably detect small (<1 cm) endometriotic deposits or the depth of infiltration. For CT, it involves irradiation and requires an intravenous contrast agent; on CT, the nodules typically appear as a solid soft tissue mass directly associated with an area of surgical scarring; and it may be difficult to distinguish scar endometriosis from bland scarring and other processes (34). MRI imaging, as the best method for describing the anatomy of a soft tissue mass and its surrounding structures, is capable of accurately defining the involvement of different anatomical structures and diagnosing the deep infiltration of abdominal and pelvic wall structures (22, 23, 35). As there were no comparative studies for different imaging modalities, we are unable to determine which imaging tool is optimal for AWE. Ultrasound, CT, and MRI, as nonspecific imaging modalities, cannot provide a definitive preoperative diagnosis. The endometriosis guidelines indicate that only histological examination can provide definitive confirmation of the diagnosis (36). Ultrasound-guided core-needle biopsy is a simple, accurate, noninvasive, easy-to-perform procedure in women with AWE, and it is useful for positive diagnosis and fordifferential diagnosis by identifying the epithelial endometrial-like cells and endometrial-like stromal cells in the lesion (7, 37, 38). In our study, all AWE patients had a history of cesarean section and a cyclic or continuous spontaneous pain. On ultrasound examination, the AWE nodules appear as heterogeneous with ill-defined, blurred outer borders, and with scarce blood vessels. The AWE nodules on MRI appear hyperintense on T1- and T2-weighted images. Furthermore, by histological examination, we found the presence of scarce endometrial glands and spindle cell stroma in AWE nodules.

The treatment goal for AWE patients is not only to relieve the abdominal cyclic pain and shrink the lesion but also to carry out a minimally invasive treatment with fewer complications. The therapeutic methods for AWE include medical management, surgical excision, and minimally invasive techniques. The success rate of medical therapy has been reported to be low, although a temporary alleviation of symptoms by using oral contraceptives, progestogens, danazol, progesterone, and aromatase inhibitors is often followed by recurrence after cessation of the drug (5, 7, 9, 10). Traditionally, surgical excision, which could offer the best chance for both definitive diagnosis and treatment, has been the primary treatment for AWE. To prevent recurrence, appropriately expanding the scope of surgical resection, such as a margin of 1 cm, is considered adequate (32). However, expanding the scope of surgical resection for AWE patients with involvement of the abdominal wall fascia and muscle may lead to complex repairs, including the need for flaps and mesh (1, 8). Recently, as a viable alternative to surgical resection, some authors have suggested that HIFU ablation and percutaneous image-guided cryoablation could reduce the lesion size, the adverse complications, and the length of hospital stay. HIFU, a conformal thermal ablation technique, can induce coagulation necrosis of the target tissues *in vivo via* ultrasound waves without injuring the adjacent normal tissues (39). A previous study showed that treatment with HIFU ablation was favorable compared to surgical resection with a shorter hospitalization time, lack of bleeding and dissemination, and fewer adverse effects (12, 39, 40). However, in most cases, scars even block acoustic beams from entering the body, causing residual lesions in the abdominal wall. Percutaneous image-guided cryoablation is performed under general anesthesia, and non-contrast CT or MRI monitoring of the growth and position of the ice ball is performed at 2–4 min intervals during the freeze–passive thaw–freeze cycle (15). Its benefits include intraprocedural ice ball visualization, which increases safety and mitigates the risk of injury to adjacent soft tissue collagenous architecture structures (14, 41, 42).

Microwave ablation, a new thermal ablation technique, has a fast heating speed, strong coagulation ability, large ablation zone, and short treatment time, and has been applied to hepatocellular carcinoma (20), thyroid carcinoma (19, 43–46), lung, kidney, and, more rarely, to the bone, pancreas, and adrenal glands (16). Currently, only one case series (*n* = 3) has reported the use of microwave ablation for the treatment of AWE (47). In our study, we used the same treatment method for AWE with a larger study population (*n* = 8) and found that microwave ablation treatment was effective at reducing the lesion size and relieving cyclic pain. No severe complications occurred during the 12 months of follow-up. In this study, we emphasize the use of ultrasound guidance and hydrodissection to protect the adjacent skin, muscle, and underlying bowel structures. The visualization of pathological biopsy and the relative placement of hydrodissection catheter or probes within the lesion were performed under ultrasound guidance, which has minimal radiation exposure. To the best of our knowledge, this is the largest collection of patients with AWE treated with microwave ablation that has been published to date.

The strengths of the present study included the strict inclusion criteria, precise and validated diagnosis of AWE, and comprehensive data analyses. However, several certain limitations should be considered in our study. First, it is a retrospective observational study, and AWE ultrasonography was performed at 12 months after the microwave ablation, but not at 1, 3, or 6 months. Second, this study was carried out in a single center, with a small number of subjects and a relatively short follow-up period. In the future, prospective, randomized, and multicenter studies with a larger number of patients and longer follow-up time are warranted.

## Conclusion

In conclusion, we demonstrated that ultrasound-guided microwave ablation can significantly reduce the AWE volume, relieve clinical symptoms, and have fewer complications for AWE patients. It may be a safe and effective technique for treating patients with symptomatic AWE.

## Data Availability

The original contributions presented in the study are included in the article/Supplementary Material, further inquiries can be directed to the corresponding author.
